# Diphtheria and Treatment

**Published:** 1882-01

**Authors:** Z. T. Magill

**Affiliations:** Lincoln, Mo.


### ARTICLE V.

**DIPHTHERIA AND TREATMENT.** By Z. T. MAGILL, M.D., Lincoln, Mo.

There has been much written and said about the above subject, yet the formidable ravages of the disease are still sweeping over our land at a frightful velocity. We go to the bedside of the little sufferer and follow carefully the treatment laid down in our best text books, yet how often, to our chagrin and dismay, the avenue of life is gradually clogged and the scene is ended. The question naturally arises, "Why do we allow so many children in the glow of beauty and health to succumb in from twenty-four to thirty hours from this fell destroyer." One reason is, the foe is upon its prey by the time we have resorted to our armamentarium: then a hand-to-hand battle ensues between life and death. We have no time to wait for the removal and prevention of ferment or spores in the fluids of the body by drugs *per orem.* In many cases, upon our first examination, the fauces, larynx and trachea are covered with a fibrinous exudation in the form of a false membrane. We must remove it (not by force), and prevent the re-formation. Many times it is beyond the reach of local applications with probang and gargles. Then the only direct resort is by inhalation. Relief is often given from slaking lime. Yet it has not been a success in my hands. Of late, carbolic acid is the sheet-anchor with me in the treatment. Having provided myself with an ordinary hose, from three to five feet long, and perhaps an inch in diameter, I place one end over the spout of a common tea-kettle, in which I place half a gallon of water and half an ounce of carbolic acid; then set the tea-kettle on the stove over a good fire, and when the water is at boiling point carry the other end of the hose under a blanket thrown over the patient's head and steam. The room should be closed. In a short time the patient will perspire freely, the sweat glands acting as a powerful emunctory and thereby eliminating the virus of the disease from the blood. If persevered in at short intervals, breathing becomes softer, and presently, after a succession of quick expulsive efforts, the patient throws off a coat or tube of false membrane. The acid vapor seems to prevent the re-formation of exudation. Whether it is owing to its local influence altogether on the mucous membrane, or to its action on the blood circulating through the lungs and the elimination of the virus by the functions of skin, I am not able to determine; probably by the combined influence. As the danger in many cases is chiefly from asthenia, I give alcoholic stimulants just short of alcoholism, and sulph. quinine in liberal doses; also good, nutritious diet.

